# Platelet-rich plasma injection in the treatment of patellar tendinopathy: a systematic review and meta-analysis

**DOI:** 10.1186/s43019-022-00151-5

**Published:** 2022-05-04

**Authors:** Apurba Barman, Mithilesh K. Sinha, Jagannatha Sahoo, Debasish Jena, Vikas Patel, Suman Patel, Souvik Bhattacharjee, Debanjan Baral

**Affiliations:** 1 Department of Physical Medicine and Rehabilitation, All India Institute of Medical Sciences, Bhubaneswar, Odisha 751019 India; 2 Department of General Surgery, All India Institute of Medical Sciences, Bhubaneswar, Odisha 751019 India

**Keywords:** Platelet-rich plasma, Patellar tendinopathy, Injections, Knee, Pain, Patella, Meta-analysis, Athletic injuries

## Abstract

**Purpose:**

The objective of the study was to assess the efficacy of autologous platelet-rich plasma (PRP) injections in the treatment of patellar tendinopathy.

**Methods:**

The PubMed, MEDLINE, EMBASE, CINAHL, and Cochrane Central Register of Controlled Trials databases were searched for clinical trials which compared PRP injection with other ‘active treatment’ interventions (‘Non-PRP’ injection and ‘No-injection’ treatments) or ‘No-active treatment’ interventions. Randomized and non-randomized clinical trials that had been published up to 15 November 2021, were included in the meta-analysis. The primary outcome, pain relief, was measured on a ‘visual analog scale.’ Secondary outcomes were knee functional activities and quality of life (QoL). The PRISMA guidelines were followed throughout the study.

**Results:**

Eight comparative studies were identified for inclusion in the meta-analysis. Assessment of these studies revealed that there were no significant differences in pain relief, functional outcomes, and QoL in the short, medium, and long term between PRP injection and Non-PRP injection interventions. Similarly, comparison of PRP injection to the No-active treatment intervention showed no differences in short- and medium-term pain relief. However, when PRP injection was compared to the No-injection treatment intervention extracorporeal shock wave therapy (ECWT), the former was found to be more effective in terms of pain relief in the medium term (mean difference [MD] − 1.50; 95% confidence interval [CI] − 2.72 to − 0.28) and long term (MD − 1.70; 95% CI, − 2.90 to − 0.50) and functional outcomes in the medium term (MD 13.0; 95% CI 3.01–22.99) and long term (MD 13.70; 95% CI 4.62–22.78).

**Conclusions:**

In terms of pain relief and functional outcomes, the PRP injection did not provide significantly greater clinical benefit than Non-PRP injections in the treatment of patellar tendinopathy. However, in comparison with ESWT, there was a significant benefit in favor of PRP injection.

**Supplementary Information:**

The online version contains supplementary material available at 10.1186/s43019-022-00151-5.

## Introduction

Patellar tendinopathy (also known as patellar tendinosis, jumper's knee, or inferior pole patellar tendinopathy) usually presents with anterior knee pain and tenderness at the inferior pole of the patella, causing significant morbidity among those participating in sports [1–5]. Repetitive tendon overload has been reported as the primary cause of patellar tendinopathy [2, 6]. Patellar tendinopathy usually has a substantial impact on daily work, delays the return to active participation in sports, and hampers overall sport performance of the affected person [7, 8]. If it is not being treated actively and appropriately, chronic patellar tendinopathy can reduce the quality of life (QoL) and force the person to retire prematurely from competitive sports.

Many persons who suffer musculoskeletal injuries, including those actively participating in sports, believe that PRP injections can promote the early recovery of ligament or tendon injuries and help them rapidly return to normal activities or competitive sports events. Platelet-rich plasma (PRP) injection has been shown to have an excellent regenerative potential to accelerate cellular remodeling and reduce the healing time in soft tissue (e.g., muscle, ligaments, and tendon) injuries [9–11]. PRP injection has anti-inflammatory, anti-nociceptive, and regenerative (proliferative and remodeling) properties [12–14].

Several studies [11, 15–22] have been conducted on PRP injection as a treatment for patellar tendinopathies, comparing its efficacy with that of ‘Non-PRP’ injections or other treatment interventions in terms of pain relief and functional improvement. A number of these studies [15, 18, 22] reported PRP injections to be superior to other treatment interventions in terms of pain relief and tissue healing properties, while the findings of other studies [16, 17] suggested the contrary. In light of the inconsistency of these study results on the efficacy of PRP injection in pain relief and knee functional outcomes, it is important to generate scientific evidence or measure the efficacy of PRP injection' treatment for patellar tendinopathy. In meta-analysis reported here, we compared the effectiveness of PRP injection for pain relief and knee functional activities with different treatment interventions (‘Non-PRP’ injection and ‘No-injection’ treatment) in the treatment of patellar tendinopathy. The results of this study will help frame therapeutic guidelines and form the basis for further research.

## Materials and methods

A comprehensive literature search of the PubMed, MEDLINE, EMBASE, CINAHL, and Cochrane Central Register of Controlled Trials) databases was conducted on 11 November 2021. The reference lists of relevant articles identified in the search were also searched for additional articles. The search strategy used in each database is reported in Additional file 1. Only articles written in English and present in the respective database up to and including 11 November 2021 were included in this review.

The review was registered in PROSPERO, the international database of prospectively registered systematic reviews (Centre for Reviews and Dissemination (CRD), University of York, York, UK) [PROSPERO: CRD42021290782] and was performed according to the PRISMA-P 2015 (Preferred Reporting Items for Systematic Reviews and Meta-Analyses) guidelines [23].

### Study and participant selection criteria

All comparative clinical trials (randomized clinical trials [RCT] and non-randomized clinical trials, both prospective and retrospective, were eligible for inclusion in this review. Clinical trials of less than 3 weeks' duration and where PRP injection was administered in the both case and control groups were subsequently excluded. Case series, case reports, and animal studies were also excluded. Two reviewers (AB, VP) independently screened the titles, who categorized the articles into included, excluded, or uncertain based solely on the title. If there was any uncertainty over eligibility, the full-text article was obtained and reviewed.

Persons aged ≥ 18 years with patellar tendinopathy who presented with anterior knee pain were eligible to be included in this review. No persons were excluded based on diagnostic criteria or stages of patellar tendinopathy.

### Interventions

This meta-analysis considered autologous PRP injection as the primary treatment for patellar tendinopathy. ‘Placebo treatment/injection’ or ‘any treatment modalities (injection/non-injection) other than PRP injection’ was considered to be a control intervention or comparator in this review. Bone-marrow aspiration concentrate, stem cells, whole blood, or conditioned serum injections were not included as experimental intervention groups. Studies were not excluded based on PRP injection doses, frequency of PRP injections, PRP separation techniques, and cellular components of PRP solution.

### Outcome measures

Outcomes (primary and secondary) were assessed at 8–12-weeks, 6 months, and 1 year, and are referred to as short-term, medium-term, and long-term outcomes, respectively.

The primary outcome was pain relief, as assessed using a visual analog scale (VAS; 10 cm).

The secondary outcomes were: (1) knee function or physical activities, as assessed by various questionnaires (e.g., the Victorian Institute of Sports Assessment-Patellar questionnaire [VISA-P; 100 points]); and (2) QoL, as assessed by the Short Form Health Survey questionnaire (SF-12; 100 points) and VAS global assessment of health (EQ-VAS; 100 points).

### Data collection and analysis

Data were extracted from the eligible studies on: study design, participants, intervention, comparators, outcome measures, adverse events or side effects, PRP preparation techniques, and characteristics of the PRP solution. Two reviewers (AB, SP) independently extracted the data from the included trials. The majority of differences and disputes regarding data extraction were resolved through discussion. A third reviewer (MKS) was asked to resolve some of the differences in opinions.

### Statistical analysis

The Review Manager 5.4 software package (The Cochrane Collaboration, Copenhagen, Denmark) [24] was used to perform all statistical analyses. All *P* values were 2-sided, and the significance level of the *P* value was fixed at < 0.05.

#### Assessment of risk of bias in included studies

Two reviewers (AB, SP) independently used the Cochrane Risk of Bias tool [25] to perform the risk-of-bias in the included clinical trials. Any disagreements were resolved by discussion with a third reviewer (MKS).

#### Measures of treatment effect

The outcome measures of pain relief, functional activities, and QoL scores were presented as continuous data. The adverse events were presented as categorical data. For continuous outcomes, the treatment effect sizes were reported either as mean differences (MD) or as the standardized mean difference (SMD). As per recommendation of the Cochrane handbook [25], a random-effect model was used for preparing the forest plot, as there could be heterogeneity among the original studies, which was not evident in the data.

#### Subgroup and sensitivity analysis

Subgroup analyses based on different control interventions were performed. Heterogeneity among the studies was explored by using the Chi^2^ and the I^2^ statistic. A sensitivity analysis was performed to determine the impact of removing one or more trials on the overall outcome result.

## Results

**Fig. 1:**
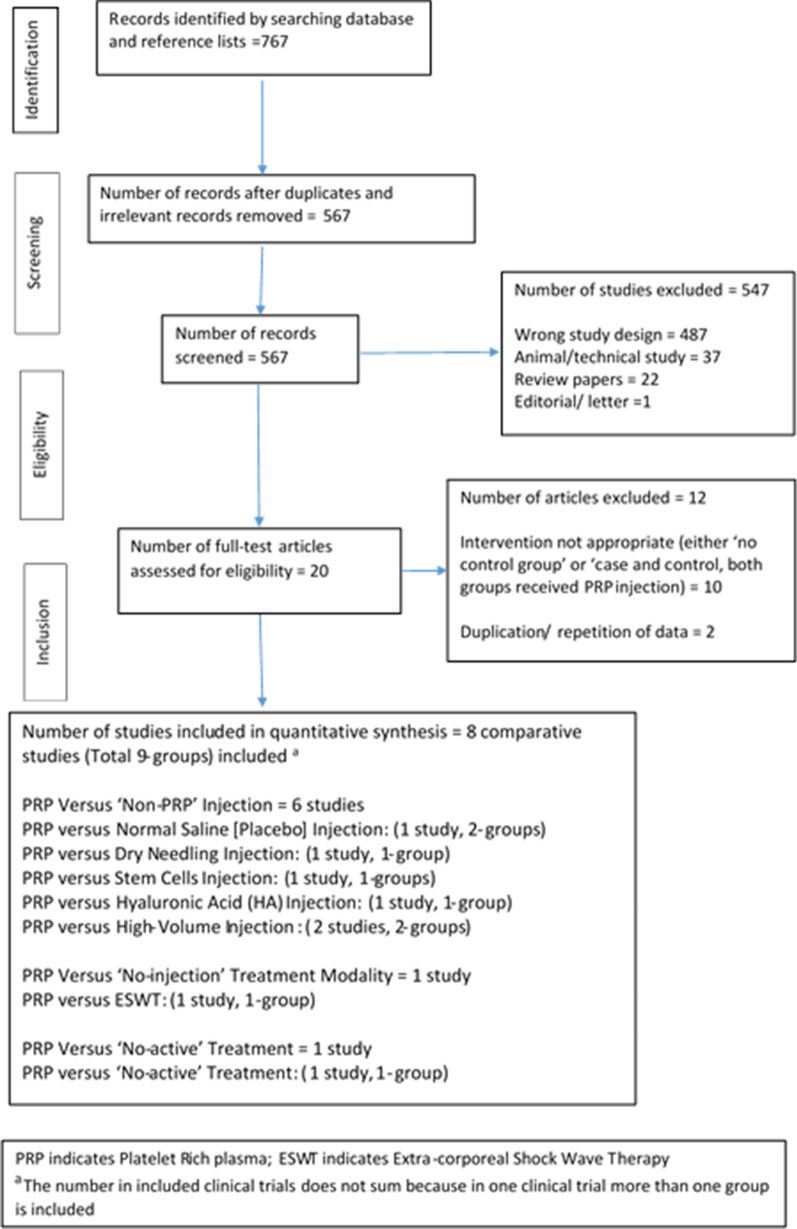
PRISMA (preferred reporting items for systematic reviews and meta-analysis) flowchart of study inclusion in the systematic literature review

A total of 767 titles were identified in the initial search. After duplicates and irrelevant articles were removed, we screened 567 articles for eligibility, identifying 20 potentially relevant full-text articles for subsequent review. Of these, 12 articles [10, 19, 26–35] (Additional file 2: Table S1) were excluded based on the inclusion and exclusion criteria. Ultimately, eight articles (5 RCTs and 3 non-RCTs; [11, 15–18, 20–22]) were included in this meta-analysis. The PRISMA flow diagram, including the reasons for exclusion, are illustrated in Fig. 1.

### Patient and study characteristics

The characteristics of the study participants are reported in Table 1. The mean age of the participants was 31.05 years, and the majority of participants were male (78%). A total of 123 persons received the PRP injection as primary treatment.

**Table 1:** Summary: the characteristics of included studies

Trial and location	Participants	Average age (years)	Male:female ratio	Design	Minimum follow-up (months)	Intervention/treatment details	PRP injection site	Co-intervention	Outcome measured	Adverse events
Scott et al. 2019 [16] Multicenter: [USA, Norway, and Italy	57 persons (athletes) with patellar tendinopathy (Blazina stage IIIB) with symptoms ≥ 6 month	32	6:1	Randomized, multicenter, prospective, single-blind	12	Group 1: Single injection of 3.5 ml of LR-PRP (USG) (*n* = 19) Group 2: Single injection of 3.5 ml of LP-PRP (USG) (*n* = 19) Group 3: Single injection of 3.5 ml of NS (USG) (*n* = 19)	Patellar tendon (at the site of the lesion)	All persons, irrespective of group, received strengthening exercises (concentric and eccentric) in a gym-based rehabilitation program: 3 times per week for 6 weeks	VISA-P (knee function and activities), NPRS (pain intensity); GRoC	No serious adverse events However, in Group 2, 1 person reported increased localized patellar tendon pain that prevented the person from participating in the rehabilitation program following injection
Dragoo et al. 2014 [17] California, USA	23 persons with patellar tendinopathy with symptoms ≥ 6 weeks	34	19:1	Randomized, single-center, prospective, double-blind	6	Group 1: Single injection of US-guided LR-PRP (6 ml) with dry needling (*n* = 10) Group 2: Single episode of US-guided dry needling (*n* = 10)	Patellar tendon (at the site of the lesion)	All persons, irrespective of group, received strengthening exercises (eccentric) and flexibility training throughout the study period	VISA (knee function and activities); VAS (pain intensity); SF-12 (QoL); Tegner scale (activity score); Lysholm scale (function and stability)	No adverse events
Vetrano et al. 2013 [15] Rome, Italy	46 persons (athletes) with patellar tendinopathy with symptoms ≥ 6 months (mean 18.9 months), un-responsive to previous non-operative treatment	26.9	4:1	Randomized, single-center, prospective, double-blind	12	Group 1: Two injections (2 ml / injection) of PRP over 2 weeks, both injections under USG (*n* = 23) Group 2: Three sessions of ESWT at 48–72 h interval under USG (*n* = 23)	Patellar tendon (at the site of the lesion)	All persons, irrespective of group, received strengthening (isometric and eccentric) and stretching exercises for 2 weeks	VISA-P (knee function and activities); VAS (pain intensity) Modified Blazina scale (treatment response)	No serious adverse events However, in Group 1, 3 persons reported increased localized patellar tendon pain and discomfort that gradually subsided following injection In Group 2, persons reported transient reddening (no bruising) of the skin following treatment sessions
Rodas et al. 2021 [20] Barcelona, Spain	20 persons with patellar tendinopathy with symptoms ≥ 4 months (mean 23.6 months) unresponsive to previous non-operative treatment	33.9 years	All males	Randomized, single-center, prospective, double-blind	12	Group 1: Two injections at an interval of 23 days [1st injection: NS; 2nd injection: BM-MSC suspended in 6 ml solution of Ringer lactate, 2% human albumin, and 5 Mm glucose) (*n* = 10) Group 2: Two injections at an interval of 23 days [6 ml each] of LP-PRP (USG) (*n* = 10)	Patellar tendon (at the site of the lesion) and peritendinous (medial and external zone)	All persons, irrespective of group, received the same post-intervention rehabilitation protocol	VISA-P (knee function and activities); VAS (pain intensity); dynamometry (muscle function);000 USG (tendon thickness and vascularity); UTC; MRI	No serious adverse events However, 1 person from each group reported increased pain (gradually subsided) following injection
Kaux et al. 2019 [18] Belgium	33 sports persons with patellar tendinopathy with symptoms > 3 months, unresponsive to previous non-operative treatment	29.4	All males	Randomized, single-center, prospective study	3	Group 1: Single injection of US-guided LP- PRP (6 ml) (*n* = 18) Group 2: Two episodes (1 week apart) of US-guided hyaluronic acid injections (*n* = 15)	Patellar tendon (at the site of the lesion)	All persons, irrespective of group, received the same rehabilitation program [strengthening and activity exercises (bicycle training)	VAS (Pain intensity); VISA-P (knee function and activities); aAlgometric scores; IKDC scores	No adverse events
Filardo et al. 2010 [19] Bologna, Italy	31 persons with patellar tendinopathy (Blazina stage IIIB) with symptoms > 3 months and unresponsive to previous non-operative treatment	27.1	All males	Non-randomized, single-center, prospective, open level	6	Group 1: Three injections (5 ml each) of PRP at an interval of 15 days (*n* = 15) and PT (exercise program) Group 2: No active interventions, only PT (exercise program) (*n* = 16)	Patellar tendon (at the site of the lesion)	All persons, irrespective of group, received the same rehabilitation program [stretching, strengthening, and activity exercises (bicycle training)	VAS (pain intensity); EQ-VAS (QoL); Tegner scale (activity score)	No adverse events
Abdelbary et al. 2018 [21] Cairo, Egypt	20 persons with patellar tendinopathy with symptoms ≥ 3 months and unresponsive to previous non-operative treatment	35.8	1:3	Non-randomized, single-center, prospective, double-blind	12	Group 1: Single injection (6 ml) of PRP under USG Group 2: Single injection of high-volume injection treatment (10 ml of 0.5% lidocaine + 25 mg hydrocortisone + 30 ml NS under USG guidance	Patellar tendon (at the site of the lesion)	All persons, irrespective of group, received the same structured rehabilitation program	VAS (pain intensity)	No adverse events
Abate et al. 2018 [22] Chieti, Italy	54 persons with patellar tendinopathy with symptoms ≥ 3 months (mean 11 months)	38.3	1:1	Non-randomized, retrospective, cohort study, open level	6	Group 1: Two injections (4-5 ml each) of PRP, at an interval of 2 weeks, under USG (*n* = 18) Group 2: Two injections of high-volume injection treatment (10 ml of 2% mepivacaine + 30 ml NS), at an interval of 2 weeks under USG (*n* = 18) Group 3: Two injections of high-volume injection treatment (10 ml of 2% mepivacaine + 30 ml NS) and 4-5 ml of PRP injections, at an interval of 2 weeks, under USG (*n* = 18)	Patellar tendon (at the site of the lesion)	All persons, irrespective of group, received the same rehabilitation program (stretching and strengthening [eccentric] for 3 months	VAS (pain intensity); VISA-P (knee function and activities);	No significant adverse

*BM-MSC* Bone marrow mesenchymal stem cells, *EQ VAS* EuroQol VAS, *GRoC* Global Rating of Change Score, *ESWT* extracorporeal shock wave therapy, *HVIGI* high-volume image-guided injection,* IKDC* International Knee Documentation Committee , *LP-PRP* leukocyte-poor PRP,* LR-PRP* leucocyte-rich PRP,* MRI* magnetic resonance imaging,* NS* normal saline, *PRP* platelet-rich plasma, *PRS* Numeric Pain Rating Scale, *PT* physio/physical therapy, *SF-12* Short Form-12 questionnaire, *USG* ultrasound guidance, *UTC* ultrasound tissue characterization,* VAS* visual analog scale, *VISA-P* Victorian Institute of Sport Assessment for Pain

The majority of studies included in this review were from European Countries. The study characteristics are reported in Table 1. The sample size of the included studies ranged from 20 to 57 participants. The VAS pain score (7 trials) [11, 15, 17, 18, 20–22] was used to measure pain relief. The Victorian Institute of Sports Assessment-Patellar questionnaire (6 trials) [15, 16, 18, 20, 22, 36] score was used to measure knee functional activities (knee and sports activities). The QoL was assessed by using the SF-12 score (1 trial) [17] and EQ-VAS score (1 trial) [11]. Three trials (4 groups) [15, 16, 21] had a follow-up duration of 1 year, and seven trials (8 groups) [11, 15–17, 20–22] had a follow-up of 6 months.

The centrifugation technique used during the PRP preparation was reported in five studies [11, 15, 20–22]. Details of the PRP preparation technique and characteristics of the PRP solution used in each study are reported in Table 2.

**Table 2:** Characteristics of the platelet-rich plasma injection used in each trial

References	PRP kit	PRP preparation	Anticoagulant used	Centrifugation technique	Leucocyte concentration in PRP solution/types of PRP (LR- or LP-PRP)	Platelets concentration (in PRP solution) (numbers × 10^3^/ml)	Increase (fold) in platelet counts (PRP) compared to baseline (whole blood)	Activating agent
Scott et al. 2019 [16]	Angel Cytomedix System (ABS-10060; Arthrex Inc, USA)	52 ml venous blood resulted in 3.5 ml PRP	Citrate dextrose Solution A	NR	LR-PRP (details not reported) LP-PRP (Details not reported)	230 × 10^3^ (LR-PRP); 227 × 10^3^ (LP-PRP)	LR-PRP: 3.8 fold LP-PRP: 3.0 fold	NR
Dragoo et al. 2014 [17]	GPS III (Biomet Inc, Warsaw, IN, USA) PRP Kit	55 ml venous blood resulted in 6 ml LR-PRP	NR	NR	LR-PRP (details not reported)	NR	NR	NR
Kaux et al. 2019 [18]	Apheresis machine (CCM TEC and Kit CS5, Fresenius-Kabi, Bad-I Iomburg, Germany)	6 ml PRP was prepared and injected	NR	NR	LP-PRP (details not reported)	850 × 10^3^ (LP-PRP)	NR	Calcium chloride
Filardo et al. 2010 [19]	NR	150 ml venous blood resulted in 20 ml PRP	NR	Double centrifugations; 1800 RPM for 15 min followed by 3500 RPM for 10 min	NR	NR	6 fold	10% Calcium chloride
Rodas et al. 2021 [20]	NR	36 ml venous blood resulted in 6 ml PRP	Citrate	Single centrifugation; 1800 RPM for 8 min	LP-PRP (details not reported)	563 × 10^3^/mm^3^	LP-PRP: 2.5 fold	5% Calcium chloride
Vetrano et al. 2013 [15]	The Recover Platelet Separation Kit (Kaylight Ltd, Israel)	10 ml venous blood resulted in 6–7 ml PRP	Acid-citrate-dextrose	Single centrifugation; 1500 RPM for 10 min	NR	0.89–1.1 × 10^9^ ml	3–5 fold	NR
Abdelbary et al. 2018 [21]	Arthrex Double Syringe System (Arthrex Inc, USA)	15 ml venous blood resulted in 6 ml of PRP	NR	Single centrifugation, 1700 RPM for 6 min	NR	NR	NR	NR
Abate et al. 2018 [22]	Regen Lab A-PRP Kit (Regenlab, Switzerland)	8 ml venous blood resulted in 6 ml of PRP	Citrate	Single centrifugation, 3400 RPM for 5 min	LP-PRP (leucocytes nil; details not reported)	NR	1.6 fold	Nil

*NR* Not reported, *RPM* revolutions per minute

**Fig. 2:**
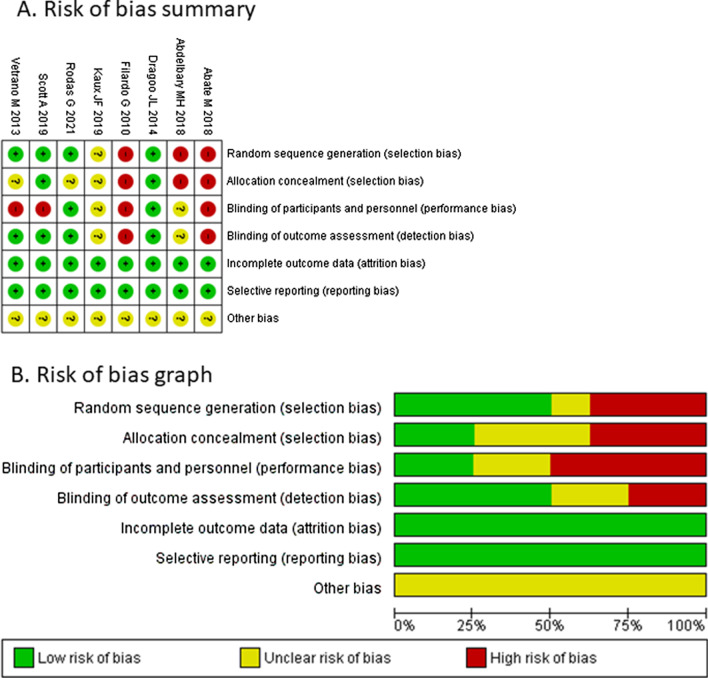
Summary of the risk of bias of each study

The graph and summary of the risk of bias of each study is shown in Fig. 2. Four (50%) trials [15, 16, 20, 36] adequately generated randomized sequence, two (25%) trials reported concealed allocation [16, 17], three (37.5%) trials blinded participants [16, 17, 20], and four(50%) trials blinded outcome assessors [15–17, 20].

### Effects of intervention

#### PRP versus non-PRP injection

Evidence from six studies (7 groups) [16–18, 20–22] suggested that there were no significant differences in pain relief (VAS pain score) and functional outcomes (VISA-P scores) in the short, medium, and long term.

**Fig. 3:**
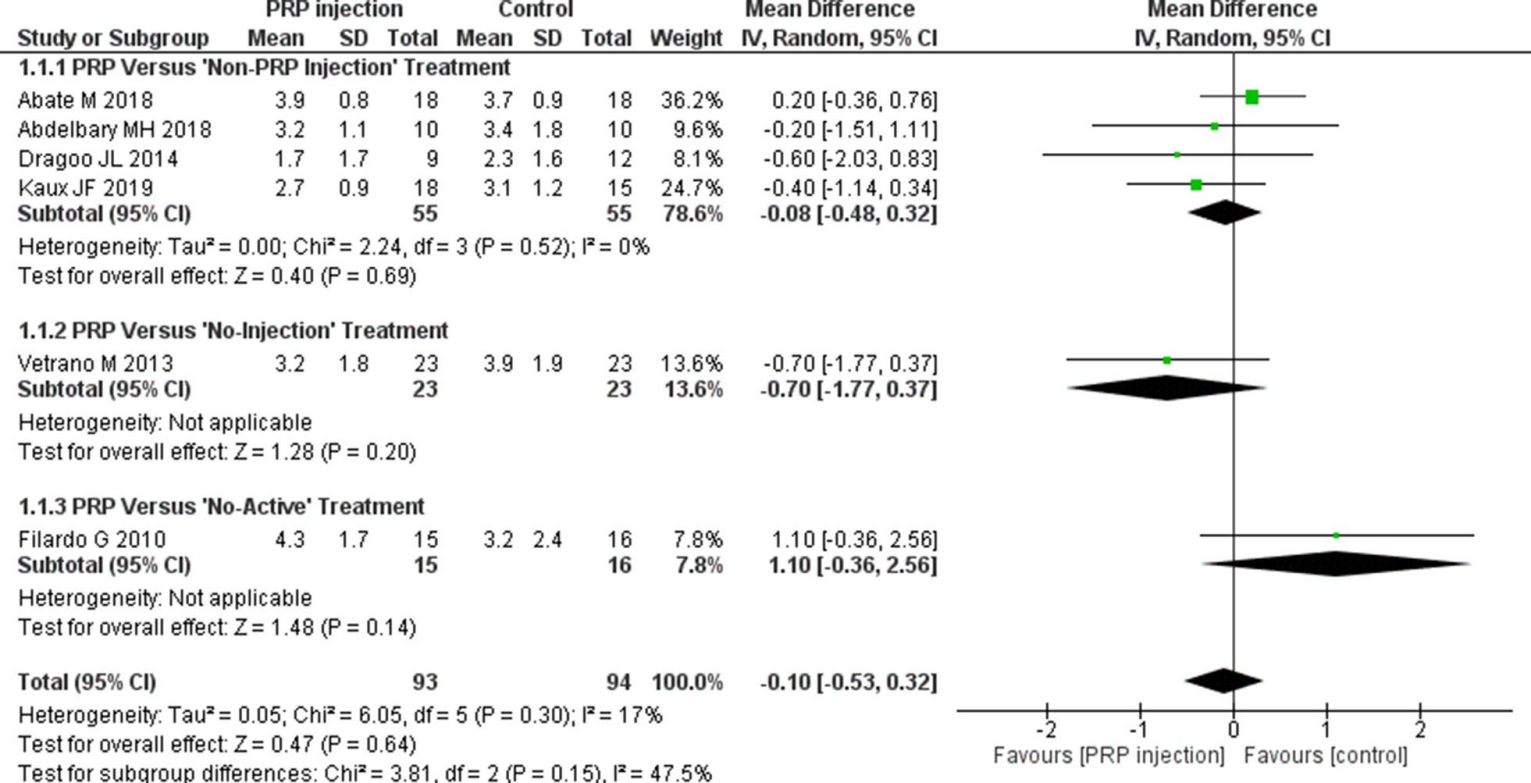
The efficacy of PRP injections (pain relief) in comparison with other interventions. Forest plot of mean improvement in pain relief (VAS pain score) in the short term (8–12 weeks).* CI* Confidence interval,* IV* weighted mean difference,* SD* standard deviation

**Fig. 4:**
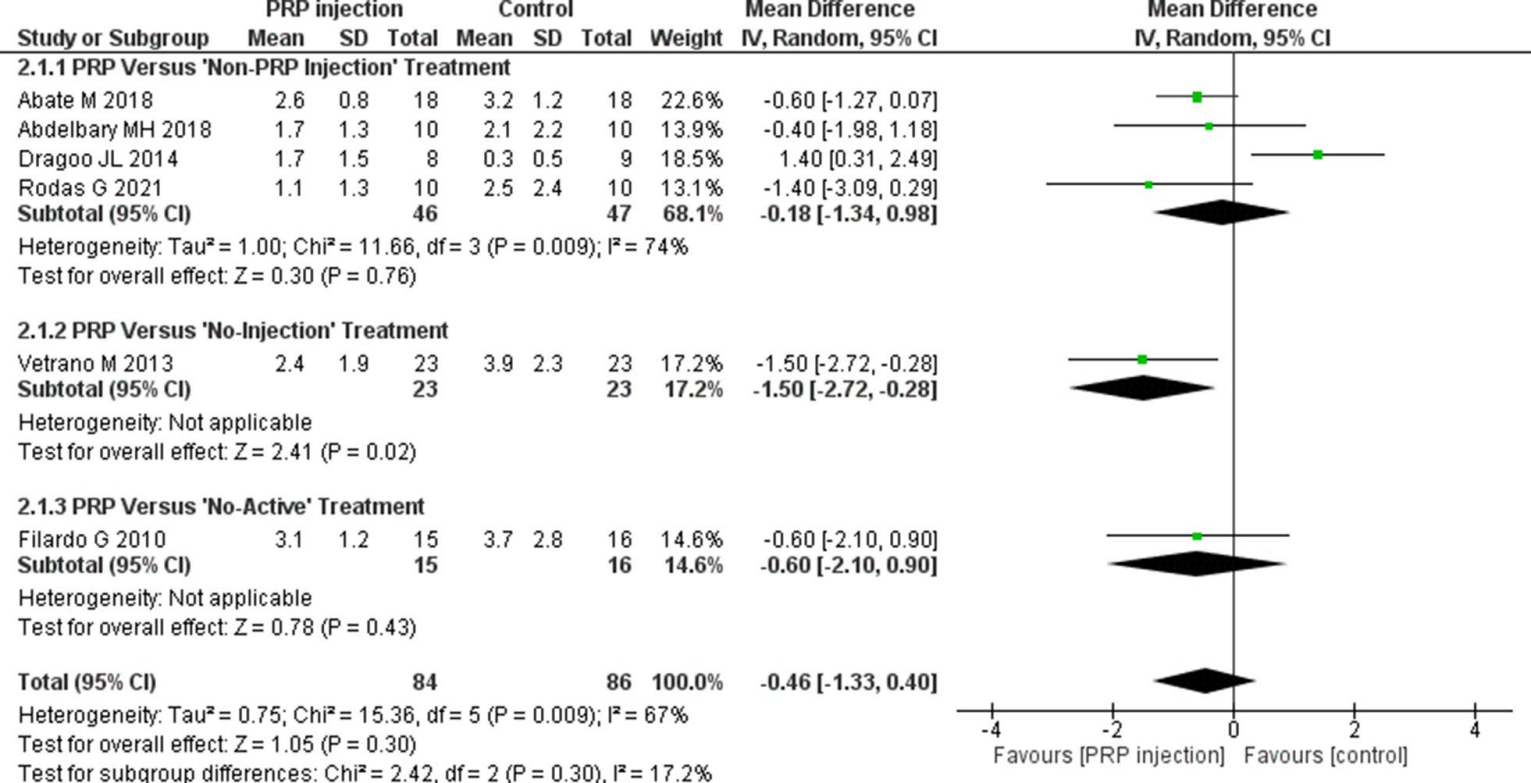
The efficacy of PRP injections (pain relief) in comparison with other interventions. Forest plot of mean improvement in pain relief (VAS pain score) in the medium term (6 months)

**Fig. 5:**
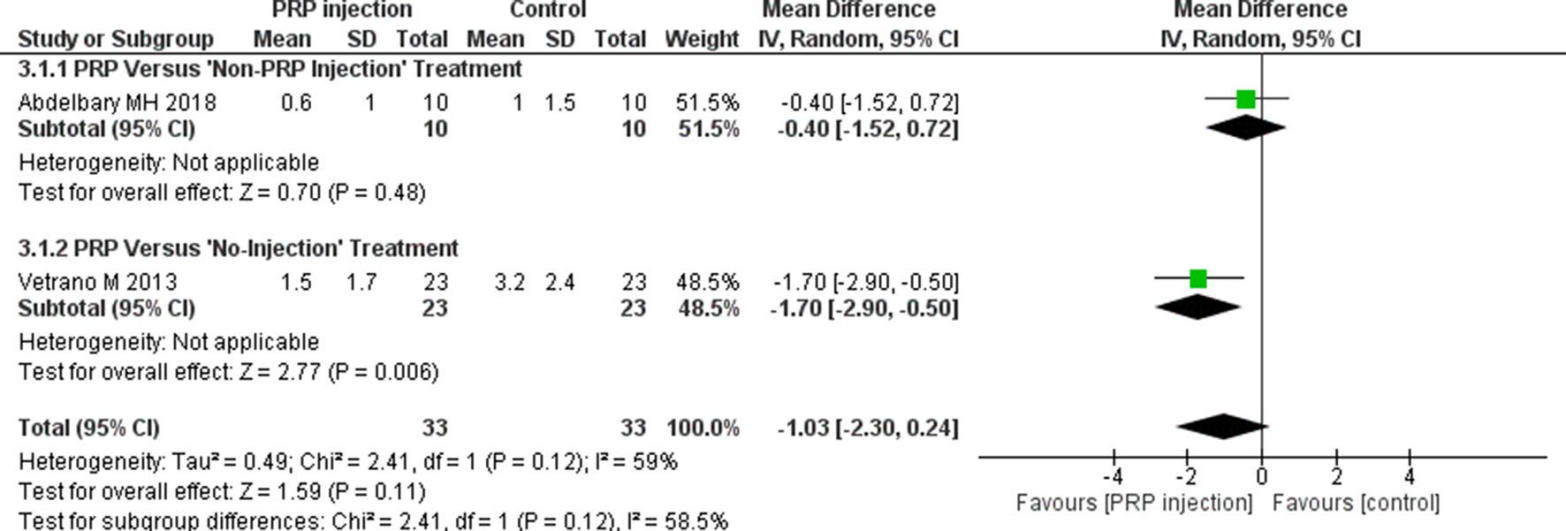
The efficacy of PRP injections (pain relief) in comparison with other interventions. Forest plot of mean improvement in pain relief (VAS pain score) in the long term (1 year)

Pain relief in the short, medium, and long term following PRP and Non-PRP injections is shown in Figs. 3, 4, and 5. The functional activities (measured by VISA-P scores) are shown in Additional file 3: Fig. S1 (short term), Additional file 4: Fig. S2 (medium term), and Additional file 5: Fig. S3 (long term)). Additional file 6: Fig. S4 shows the QoL outcomes in the short (Additional file 6: Fig. S4A) and medium term (Additional file 6: Fig. S4B). None of the studies assessed QoL in the long term.

##### PRP versus normal saline injection

 One trial [16] (level of evidence: 1) was available that compared the efficacy of PRP injection (leucocyte-rich PRP [LR-PRP] and leucocyte-poor PRP [LP-PRP]) with normal saline (NS) (placebo) injection. The authors of this study administered a single injection of PRP (LR-PRP or LP-PRP) into two different groups, with one group (*n* = 19) receiving LR-PRP injection and the second group (*n* = 19) receiving LP-PRP injection, and then compared the efficacies of these treatments with another group who received NS injections (*n* = 19). All subjects (*n* = 57), irrespective of their allocated groups, followed a supervised gym-based rehabilitation program following the respective intervention (LR-PRP, LP-PRP and NS injections). The follow-up was 1 year. The results showed that compared to NS injection, persons who received PRP injections (LR-PRP or LP-PRP) did not demonstrate a significant benefit in functional activities (VISA-P scores) in the short (Additional file 3: Fig. S1), medium (Additional file 4: Fig. S2), and long term (Additional file 5: Fig. S3). Rather, at the 1-year (long-term) follow-up, persons who received NS injections showed a more remarkable improvement in VISA-P score (Additional file 5: Fig. S3). In this study [16], the pain was not assessed with the VAS pain scores; pain relief was measured with a numerical pain rating scale (NRS). The NRS pain scores were not different among the three groups (LR-PRP, LP-PRP, and NS) at any of the follow-up visits (short, medium, and long term). The numerical pain rating scale scores were not included in the pooled data analysis, where pain relief was measured with VAS pain scores.

##### PRP versus dry needling injection

 Dragoo et al. [17] conducted a RCT (level of evidence: 1) which compared the efficacy of a single injection of LR-PRP (*n* = 10) with a single episode of dry needling (DN) intervention (*n* = 12). Following the interventions, all subjectss (*n* = 22) followed a supervised, structured exercise program (eccentric strengthening exercises, flexibility, cardiovascular, balance training, core strengthening exercises, and sport-specific skills). The follow-up was 1 year.

 Evidence from this one RCT [17] indicated that those subjects who received DN injections demonstrated more significant pain relief (MD 1.40, 95% confidence interval [CI] 0.31–2.49) (Fig. 4) in the medium term. No difference was observed in short-term pain relief. In terms of functional activities (VISA-P scores), there was a tendency to improved functional outcomes in favor of PRP injections at both follow-up visits (short term [Additional file 3: Fig. S1; medium term [Additional file 4: Fig. S2]), but no significant differences (VISA-P scores) were found between these two interventions. Similarly, in terms of QoL (Additional file 6: Fig. S4), there were no differences between the groups (PRP vs. DN) in the short and medium term.

##### PRP versus stem cell injection

 Rodas et al. [20] conducted a RCT (level of evidence: 2) which compared the efficacy of a single injection of PRP (*n* = 10) with a single injection of stem cells (bone marrow-derived mesenchymal stem cells) (*n* = 10) in chronic patellar tendinopathy (lesion size > 3 mm). The study duration was 6 months. At the end of the study (6 months), although there was a tendency of increased pain relief in favor of the PRP group, there were no significant differences between these two groups (Fig. 4). Similarly, in knee functional activities (VISA-P scores), there was no substantial difference between the groups (Additional file 4: Fig. S2).

##### PRP versus high-volume image-guided injections

Two studies [21, 22] compared the efficacy of PRP injection (*n* = 28) with high-volume image-guided injections (HVIGI) (*n* = 28). In their study on HVIGI (level of evidence: 2), Abdelbary [21] used hydrocortisone (25 mg) together with 30 ml NS in HVIGI (*n* = 10). In contrast, in their study (level of evidence: 3), Abate et al. [22] administered 30 ml of NS (without hydrocortisone) (*n* = 18). Abdelbary et al. [21] administered only one PRP injection, while Abate et al. [22] repeated the injections (PRP or HVIGI) in the same knee after 2 weeks. In both studies, injections were administered at the interspace between the fat pad and the patellar tendon of the target knee joint. Pooled analysis from these two studies [21, 22] showed increased pain relief (albeit no significant difference) with PRP injection in the medium term (MD − 0.57, 95% CI − 1.18 to 0.04). Knee functional outcome (VISA-P score) was measured only in one study [22]. Abate et al. [22] found no significant difference in short-term functional outcome between the two groups, but they did find a highly significant VISA-P score with PRP injection in the medium-term follow-up visit (Additional file 4: Fig. S2). Abdelbary et al. [21] did not assess knee functional outcome (VISA-P).

##### PRP versus hyaluronic acid injections

Kaux et al. [18] conducted a study (level of evidence: 2) in which they compared the efficacy of a single injection of PRP (LP-PRP) (*n* = 18) with two injections of hyaluronic acid (HA) (administered 1 week apart) (*n* = 15) at 3 months. No significant differences in VAS pain relief (Fig. 3) and VISA-P scores (Additional file 3: Fig. S1) were noted at 3 months. However, there was a trend of improved pain relief (3 months) in favor of PRP injection (Fig. 3).

#### PRP versus No-injection treatment modalities

##### PRP versus extracorporeal shock wave therapy

 One RCT [15] (level of evidence: 1) was included in this review that compared the efficacy of PRP injection with extracorporeal shock wave therapy (ESWT). The authors of this study divided 46 persons with patellar tendinopathy equally into two groups, with one group (*n* = 23) treated with two PRP injections (1-week interval) and the second group (*n* = 23) treated with three sessions of ESWT (each session comprising 2,400 impulses at 0.17–0.25 mJ/mm^2^) at intervals of 48 to 72 h. Study participants were followed up at 2, 6, and 12 months. All subjects followed a structured exercise program comprising stretching (knee-flexors, extensors, hip flexors, and tendoachillis [TA]) and strengthening (isometric and isotonic exercises) exercises for 2 weeks. Those persons who received PRP injections showed more significant improvements (*P* < 0.05) in terms of VAS pain scores at both visits (medium term [Fig. 4] and long term [Fig. 5]). Similarly, in terms of functional activities (VISA-P scores), persons in the PRP group achieved much better scores (*P* < 0.05) in the medium term (Additional file 4: Fig. S2) and long term (Additional file 5: Fig. S3).

#### PRP versus No-active treatment

One study was found in which the efficacy of PRP injection was compared with No-active treatment interventions. In this study (level of evidence: 2), Filardo et al. [11] recruited 31 persons with patellar tendinopathy (with grade III-b [Blanzina criteria]). These authors administered three PRP injections (at 2-week intervals) at the lesion sites (*n* = 15) and compared its efficacy with 16 persons with patellar tendinopathy who received No-active treatment. Both groups received formal exercise therapy, the same exercise therapy as at home. The study duration was 6 months. At the end of the study, the authors found no greater improvements (*P* > 0.05) in pain relief (Figs. 3, 4) and QoL (Additional file 6: Fig. S4) in the intervention group.

### Safety outcomes

None of the studies [11, 15–18, 20–22] reported significant adverse events following PRP injection. However, the authors of three studies [15, 16, 20] reported increased pain (localized patellar tendon pain) following LP-PRP injection. Scott et al. [16], Vetrano et al. [15], and Rodas et al. [20] reported one (10%), one (5%), and three (13%) patients, respectively, who complained of increased local pain following LP-PRP injection [15, 16, 20]. In all three studies, the pain, which started following injections, subsided with time.

## Discussion

After an extensive literature search, we identified eight studies that met our inclusion criteria and were eligible for inclusion in this review. These studies demonstrated no differences in pain relief and functional outcomes between PRP injections and Non-PRP injections. Compared to NS injection, PRP injection did not provide additional benefit in knee function and knee activities up to 1 year of follow-up. Compared with the No-active treatment intervention, PRP injection did not significantly reduce pain at 3 and 6 months. PRP injection was found to be superior in terms of knee pain and functional activities (in the short and medium terms) only when it (PRP injection) was compared with the No-injection treatment modality ESWT.

Exercise training, especially eccentric-strength training, provides a greater mechanical stimulation at the injured site. Eccentric-strength training activates the tendon stem/progenitor cells (TSPCs) in increased numbers, which helps tissue healing and improves tissue metabolism at the injured site [2, 22, 37–40]. Studies [37, 38, 40] have reported that knee-extensor strengthening exercises, especially high-load eccentric strengthening exercises, can improve knee pain and functional activities in persons with patellar tendinopathies. In this review, we noted that all studies included structured knee exercise programs (including eccentric strengthening exercises) as ‘Co-interventions’ with PRP injection. In their study, Filardo et al. [11] even failed to demonstrate additional benefit (in terms of pain relief) with three consecutive injections of PRP. Therefore, it is challenging to speculate that the improvements observed in a few studies [15, 20, 22, 36] after PRP injections were due to PRP injection, and not to the structured exercise program.

Patellar tendinopathy is common among persons who are active in sports activities, particularly in those sports that involves frequent jumping, such as volleyball, basketball, and soccer [11, 15, 41]. The prevalence of patellar tendinopathy among elite athletes can reach 14%, increasing with the duration of time the person actively participates (up to 22%) [41]. A complete recovery (no pain even after extensive sports injury) or a return to competitive sports are the primary goals of any person affected by a sports injury. It is crucial to judge the efficacy of the intervention (including PRP injection) among sportspersons in terms of complete recovery/return to competitive sports. We found only two studies [11, 15] in which the authors reported the efficacy of PRP injections in achieving the states of complete recovery/return to competitive sports. Pooled data from these two studies [11, 15] showed that at the 6-month follow-up there were no significant differences in complete recovery/return to competitive sports (risk ratio 1.26, 95% CI 0.59 to 2.69, *P* = 0.55] between PRP injection and the control interventions (ESWT and No-active treatment).

Although there is consensus among researchers that increased growth factors and platelets in the PRP solution increase tissue healing and regenerative properties, there is still controversy regarding the presence of leucocytes in the PRP solution. It has been reported [42] that the presence of leucocytes in PRP favors early recovery (due to increased inflammation) in tissue healing. In contrast, a few studies have reported that the presence of leucocytes in PRP may delay tissue healing. Leucocytes in the PRP injection may release the matrix metalloprotease and reactive oxygen, slowing the healing time or damaging the tissues [14, 43]. Similarly, there is no standard protocol for the PRP preparation technique [14]. Studies have reported different PRP preparation techniques (centrifugation, rate, duration). Depending on the PRP preparation procedure (centrifuge machine, speed, duration and number of centrifugations, duration), cell components of PRP can be different [14, 44]. Regarding the application of PRP injection in musculoskeletal injuries, there is no standard guideline on the volume to be injected, injection method, and injection frequency. Therefore, before any conclusion can be drawn or the quality of PRP research assessed, it is essential to standardize the PRP solution (minimal critical components in blood components in PRP), PRP preparation techniques, PRP injection dose, and frequency.

The present meta-analysis differs from the previously conducted systematic reviews [2, 3, 14, 42]. Our study failed to demonstrate significant improvement over other treatment modalities in pain relief and knee functional activities following PRP application. The current meta-analysis included many clinical trials (8 comparative studies), compared to Dupley et al. [3] who included only two clinical trials in their meta-analysis. In their reviews, Matteo et al. [42] included all lower limb tendinopathies (patellar and Achilles tendinopathies), but they [42] did not conduct a quantitative analysis. Andriolo et al. [2], in their review, included all kinds of studies, including case series and comparative studies, where PRP injection was also administered in the control group. In their systematic review, Jeong et al. [14] included articles with all study designs (including case reports, and retrospective studies) and did not conduct quantitative analysis in their reviews.

There are a number of strengths to this review. First, we conducted a comprehensive literature search. All comparative clinical trials available in an electronic database were included in this review. Second, to address the methodological differences between studies, we performed subgroup analyses. Third, short-, medium-, and long-term efficacies were assessed. Most of the participants were followed up for 1 year.

It is important to note that there are alsoe some limitations to this review. First, the total number of participants in each study was significantly low. A variety of other (control) interventions and PRP preparation techniques were used in the included trials. The control group's treatment modalities (placebo, DN, stem cell, HA, ESWT, and No-active treatment) were heterogeneous. Second, although eight articles were included, most of the findings in the subgroup analysis were based only on one clinical trial. Therefore, we should carefully explain the effect sizes of pain relief and knee functional outcomes because further research might change the impact of these estimates. Third, none of the studies reported individual participants’ performance in sports events, or ultra-sonographic findings following PRP injection. Pain relief and the functional outcome might not be equivalent to a “return to sports” or “enhanced sports performance.” Fourth, the studies included in the analysis had methodological limitations. Both prospective and retrospective clinical trials were included. Proper concealments and blinding were not performed in many studies, including in RCTs. A few studies compared PRP injections with No-injection or No-active treatment techniques. None of the studies reported growth factors. Therefore, it is necessary to consider all these factors when interpreting the results.

## Conclusions

In terms of pain relief and functional outcomes, the PRP injection did not provide significantly greater clinical benefit than Non-PRP injections in patellar tendinopathy. However, in comparison with ESWT, there was a significant benefit in favor of PRP injection. Based on these findings, we cannot recommend for or against the PRP injection in the management of patellar tendinopathy until more homogenous clinical trials or a more robust, high-quality RCT is available.

## Supplementary Information

**Additional file 1. **Literature search strategies.

**Additional file 2: Table S1. **Excluded studies.

**Additional file 3: Fig. S1.** The efficacy of PRP (platelet-rich plasma) injections (knee function and activities) in comparison with other interventions. Forest plot of mean improvement in VISA-P (Victorian Institute of Sports Assessment-Patellar questionnaire) in the short term (8–12 weeks).

**Additional file 4: Fig. S2.** The efficacy of PRP injections (knee function and activities) in comparison with other interventions. Forest plot of mean improvement in VISA-P in the medium term (6 months).

**Additional file 5: Fig. S3.** The efficacy of PRP injections (knee function and activities) in comparison with other interventions. Forest plot of mean improvement in VISA-P in the long term (1 year).

**Additional file 6: Fig. S4.** The efficacy of PRP injections quality of life (QoL) in comparison with other interventions. Forest plot of mean improvement in QoL.** A** Short term (8–12 weeks),** B** medium term (6 months).

## Data Availability

All data generated or analyzed during this study are included in this published article.

## Abbreviations

CI: Confidence interval
DN: Dry needling
ESWT: Extracorporeal shock wave therapy
HA: Hyaluronic acid
LP-PRP: Leucocyte poor platelet-rich plasma
LR-PRP: Leucocyte rich platelet-rich plasma
MD: Mean difference
NS: Normal saline
PRP: Platelet-rich plasma
QoL: Quality of life
RCT: Randomized controlled trial
VAS: Visual analog scale
VISA-P: Victorian Institute of Sports Assessment–Patellar questionnaire
