# Supplementary material for: Platelet-rich plasma injection in the treatment of patellar tendinopathy: a systematic review and meta-analysis
Source: Knee Surg Relat Res. 2022 May 4;34:22. doi: 10.1186/s43019-022-00151-5 (PMC9066802; doi:10.1186/s43019-022-00151-5)
Supplement: Supplementary file 1 — Additional file 1. Literature search strategies. [file 43019_2022_151_MOESM1_ESM.docx]

**Literature Search Strategies**

Identification of trials and search method

Text words with key terms for electronic database

1. **PubMed**

---------------------------------------------------------------------------------------------------------------------------------

1. “Tendinopathy” [MeSH]
2. “patellar tendinopathy”[Title/ Abstract] OR “patellar tendinosis”[Title/ Abstract] OR OR “jumper’s knee”[Title/ Abstract] OR patella [Title/ Abstract]
3. #1 OR #2
4. Platelet-Rich Plasma[MeSH] OR "Blood Platelets "[MESH] OR Platelet-Derived Growth Factor[MeSH] OR Platelet-Rich Fibrin[MeSH]
5. “platelet rich plasma”[Title/ Abstract] OR “platelet concentrate”[Title/ Abstract] OR “PRP”[Title/ Abstract] OR “PDGF”[Title/ Abstract] OR “platelet plasma”[Title/ Abstract] OR “plasma growth factors”[Title/ Abstract] OR “autologous”[Title/ Abstract] OR “autologous blood”[Title/ Abstract] OR “autologous conditioned plasma”[Title/ Abstract] OR platelet gel”[Title/ Abstract] OR platelet-derived OR “platelet-rich fibrin”[Title/ Abstract])
6. #4 OR #5
7. #3 AND #6
8. **Cochrane Central Register (Cochrane Library via Wiley)**

----------------------------------------------------------------------------------------------------------------------------

1. MeSH descriptor: [Tendinopathy] explode all trees
2. (“patellar tendinopathy”): ti, ab, kw OR (“patellar tendinosis”): ti, ab, kw OR (“jumper’s knee”): ti, ab, kw OR ("Patella"): ti, ab, kw
3. #1 OR #2
4. MeSH descriptor: [Platelet-Rich Plasma] explode all trees
5. MeSH descriptor: [Platelet-Derived Growth Factor] explode all trees
6. MeSH descriptor: [Platelet-Rich Fibrin] explode all trees
7. MeSH descriptor: [Blood Platelets] explode all trees
8. (“platelet rich plasma”): ti, ab, kw OR (“platelet concentrate”): ti, ab, kw OR (“PRP”): ti, ab, kw OR (“PDGF”): ti, ab, kw OR (“platelet plasma”): ti, ab, kw OR (“plasma growth factors”) ti, ab, kw OR (“autologous”): ti, ab, kw OR (“autologous blood”): ti, ab, kw OR (“autologous conditioned plasma”): ti, ab, kw OR (“platelet gel”): ti, ab, kw OR (“platelet-derived”): ti, ab, kw OR (“platelet-rich fibrin”): ti, ab, kw
9. #4 OR #5 OR #6 OR #7 OR #8
10. #3 AND #9
11. **CINAHL (EBSCO)**

-----------------------------------------------------------------------------------------------------------------------------

S17 S7 AND S16

S16 S8 OR S9 OR S10 OR S11 OR S12 OR S13 OR S14 OR S15 OR S15

S15 MH “autologous conditioned plasma”

S14 MH “platelet derived growth factor”

S13 MH “platelet gel”

S12 MH “platelet rich plasma injection”

S11 MH “platelets”

S10 MH “platelet rich fibrin”

S9 MH “platelet rich plasma therapy”

S8 MH “platelet rich plasma”

S7 S1 OR S2 OR S3 OR S4 OR S5 OR S6

S6 MH “jumper's knee”

S5 MH “patella”

S4 MH “patellar tendinitis”

S3 MH “patellar tendinopathy”

S2 MH “tendinopathy or tendonitis or tendonopathy or tendinosis or tendinosis”

S1 MH “tendinopathy”

1. **MEDLINE (Ovid)**

-------------------------------------------------------------------------------------------------------------------------------

1. ‘tendinopathy’/exp
2. ‘patella’/exp
3. ‘tendinopathy’: ab, ti OR ‘patella’: ab, ti OR ‘jumper’s knee’: ab, ti OR ‘tendinosis’: ab, ti /
4. #1 OR #2 OR #3 OR #4
5. ‘thrombocyte rich plasma’/exp OR ‘platelet-derived growth factor’/exp OR ‘thrombocyte’/exp
6. ‘platelet rich plasma’: ab, ti OR ‘platelet concentrate’: ab, ti OR ‘prp’: ab, ti OR ‘pdgf’: ab, ti OR ‘platelet rich therapy’ ab, ti OR ‘autologous’: ab, ti OR ‘autologous blood’: ab, ti OR ‘autologous conditioned plasma’: ab, ti OR ‘platelet gel’: ab, ti OR ‘platelet derived growth factors’: ab, ti OR ‘platelet-rich fibrin’: ab, ti
7. #5 OR #6
8. #4 AND #7
