# Supplementary material for: Platelet-rich plasma injection in the treatment of patellar tendinopathy: a systematic review and meta-analysis
Source: Knee Surg Relat Res. 2022 May 4;34:22. doi: 10.1186/s43019-022-00151-5 (PMC9066802; doi:10.1186/s43019-022-00151-5)
Supplement: Supplementary file 2 — Additional file 2: Table S1. Excluded studies. [file 43019_2022_151_MOESM2_ESM.docx]

**Table S1:** Excluded studies

| **No** | **Study** | **Reason for exclusion** |
| --- | --- | --- |
| 1 | Gosens 2012 [10] | Case-control, both groups received PRP injections. |
| 2 | Filardo 2013 [19] | Prospective case series, no comparator group. |
| 3 | Zayni 2015 [26] | Case-control, both groups received PRP injections. |
| 4 | Kaux 2015 [27] | Prospective case series, no comparator group. |
| 5 | Kaux 2016 [28] | Case-control, both groups received PRP injections (single versus multiple PRP injections) |
| 6 | Ferrero 2012 [29] | Prospective case series, no comparator group. |
| 7 | Van Ark 2013 [30] | Prospective case series, no comparator group. |
| 8 | Manfreda F 2019 [31] | Prospective case series, no comparator group. |
| 9 | Kirschner JS 2020 [32] | RCT on chronic tendinosis, no separate analysis was done for patellar tendinopathy |
| 10 | Barry HC 2019 [33] | Duplication/ repetition of data: Synopsis of the article, published by Scott A et. Al. 2019 |
| 11 | Smith J 2014 [34] | Duplication/ repetition of data: Commentary on the article, published by Vetrano M et. Al. 2013 |
| 12 | Charousset 2014 [35] | Prospective case series, no comparator group. |
