# Supplementary figures and images for: Platelet-rich plasma injection in the treatment of patellar tendinopathy: a systematic review and meta-analysis
Source: Knee Surg Relat Res. 2022 May 4;34:22. doi: 10.1186/s43019-022-00151-5 (PMC9066802; doi:10.1186/s43019-022-00151-5)

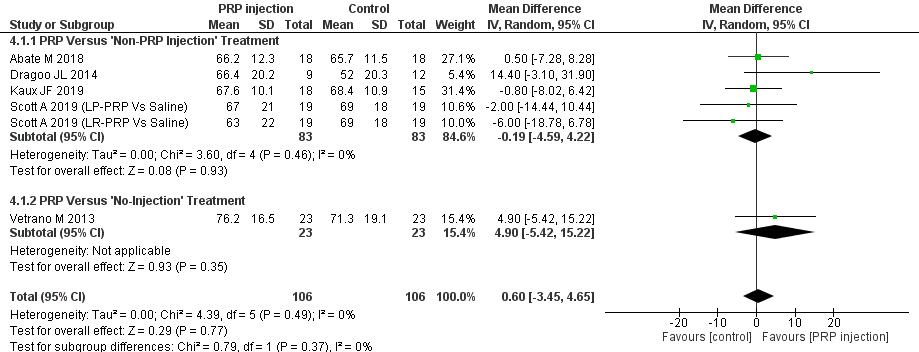

Supplement: Supplementary file 3 — Additional file 3: Fig. S1. The efficacy of PRP (platelet-rich plasma) injections (knee function and activities) in comparison with other interventions. Forest plot of mean improvement in VISA-P (Victorian Institute of Sports Assessment-Patellar questionnaire) in the short term (8–12 weeks). [file 43019_2022_151_MOESM3_ESM.tiff]

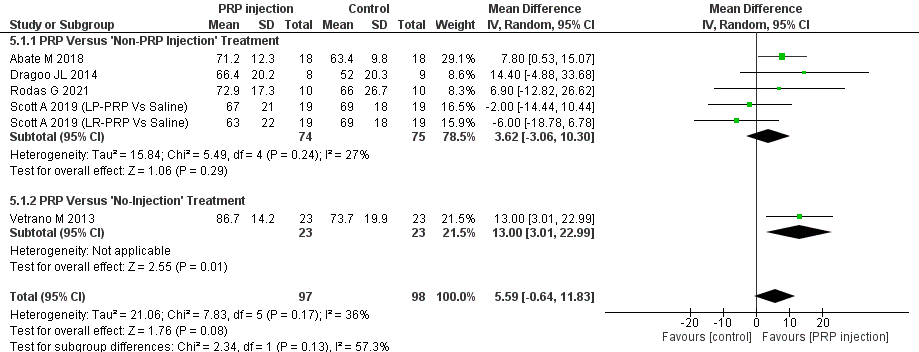

Supplement: Supplementary file 4 — Additional file 4: Fig. S2. The efficacy of PRP injections (knee function and activities) in comparison with other interventions. Forest plot of mean improvement in VISA-P in the medium term (6 months). [file 43019_2022_151_MOESM4_ESM.tiff]

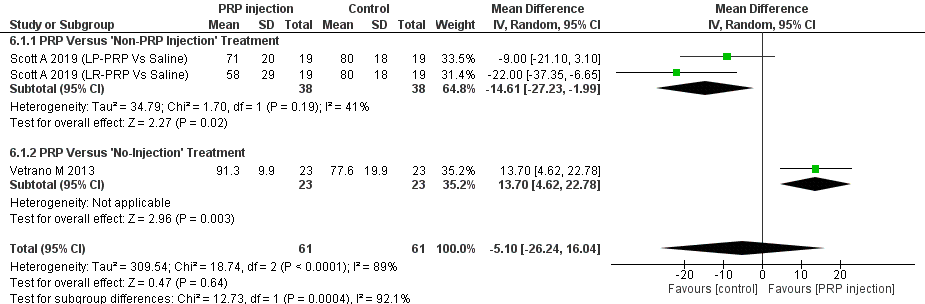

Supplement: Supplementary file 5 — Additional file 5: Fig. S3. The efficacy of PRP injections (knee function and activities) in comparison with other interventions. Forest plot of mean improvement in VISA-P in the long term (1 year). [file 43019_2022_151_MOESM5_ESM.tiff]

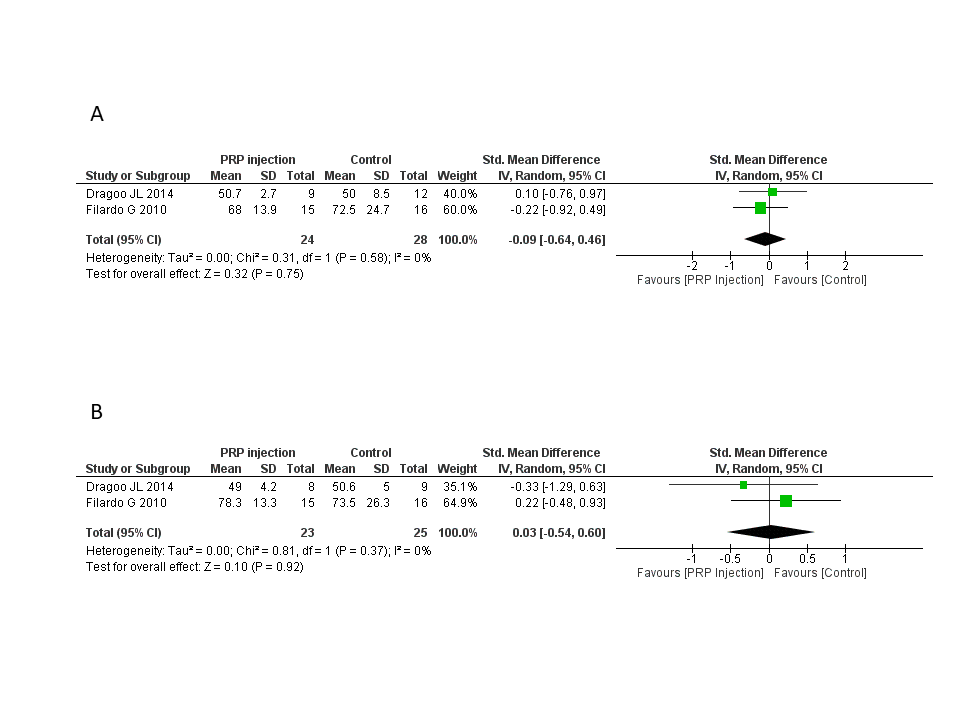

Supplement: Supplementary file 6 — Additional file 6: Fig. S4. The efficacy of PRP injections quality of life (QoL) in comparison with other interventions. Forest plot of mean improvement in QoL. A Short term (8–12 weeks), B medium term (6 months). [file 43019_2022_151_MOESM6_ESM.tif]
